# Prevalence and determinants of burnout syndrome among physicians in Cameroon: a research proposal

**DOI:** 10.1186/s13104-017-2833-0

**Published:** 2017-10-25

**Authors:** Vitalis Fambombi Feteh, Tsi Njim, Miriam A. M. Nji, Chia Mark Ayeah, Carlson-Babila Sama, Frank Leonel Tianyi

**Affiliations:** 1 Mboppi Baptist Hospital Douala, P.O. Box 15161, Douala, Cameroon; 2 Health and Human Development (2HD) Research Network, Douala, Cameroon; 3 Centre for Global Health and Tropical Medicine, Nuffield Department of Medicine, University of Oxford, Oxford, UK; 4 Sub-Divisional Hospital Nkwen, Bamenda, Cameroon; 5 School of Life and Medical Sciences, Faculty of Population Health Sciences, Institute for Global Health, University College London, London, UK; 6 Galactic Corps Research Group (GCRG), Buea, Cameroon; 7 Sub-Divisional Hospital Mayo Darley, Mayo Darley, Cameroon

**Keywords:** Burnout, Physician, Prevalence, Determinants, Cameroon, Maslach Burnout Inventory

## Abstract

**Objectives:**

Burnout syndrome is a common psychological state, that may affect human healthcare providers due to their prolonged exposure to job stressors. Burnout can hinder optimal healthcare delivery. Hence this study aims to determine the prevalence and correlates of burnout syndrome amongst physicians in Cameroon. Specifically: (1) to determine the prevalence of burnout syndrome amongst Cameroonian doctors. (2) To identify potential determinants of burnout among Cameroonian doctors. (3) To compare the prevalence and determinants of burnout among specialist physicians and general practitioners in Cameroon.

**Results:**

This cross-sectional study will include a minimum of 335 doctors working in five regions of Cameroon. Consenting physicians will be consecutively recruited and data on sociodemographic and work characteristics will be collected via a printed self-administered questionnaire and burnout will be assessed using the Maslach Burnout Inventory. Data will be analysed using Epi Info version 7 and a p value < 0.05 will be considered significant. Multivariable logistic regression will be used to identify determinants of burnout syndrome. Physicians’ mental health is largely neglected in developing countries like Cameroon. Data from this research will help inform practitioners on the magnitude of the problem and favour the development of policies that improve the mental health of care-providers.

## Introduction

Burnout syndrome is a psychological state induced by prolonged exposure to job-related stressors [1]. It has three main features: emotional exhaustion (referring to the feelings of being emotionally overworked), depersonalization (relating to an impersonal response towards recipients of one’s service) and personal accomplishment (standing for feelings of competence and successful achievement in one’s work with people)—which is negatively affected as the burnout continuum progresses [2]. According to the World Health Organization’s (WHO) International Classification of Diseases (ICD), 10th revision, burnout (coded as “Z73.0, Problems related to life management difficulty”) is defined as a “state of vital exhaustion” and it includes mental and physical exhaustion related to stress at work [3].

Burnout is increasingly getting recognition in the sphere of health providers’ health, as it is very common amongst medical personnel with a prevalence ranging from 27 to 75% [4] and with an increasing trend among American physicians, compared to the general population [5]. It has been postulated that burnout starts at the early stages of medical training and often worsens during practice [6, 7].

Physician’s burnout has severe consequences on the outcomes for patients, the performance of health institutions as well as physician’s sense of wellness [8]. Doctors experiencing burnout are more likely to make poor decisions and more medical errors; display hostile attitude towards patients; and have difficult working relationships. Also, burnout increases risk of depression; anxiety; sleep disturbances; fatigue; alcohol and drug misuse; marital dysfunction; premature retirement and even suicide [9]. In developing countries, adverse working conditions and lack of career advancement options may exacerbate the occurrence of burnout in doctors [10].

Cameroon has a population of about 24 million inhabitants, with a physician density of approximately 1:12,500 people [11]. However, the nationwide distribution of doctors is so inhomogeneous, for instance, a physician in the Center Region caters for 5449 persons while a physician in the Adamawa Region caters for 26,726 persons [12]. It is thus plausible that the density of specialists in Cameroon will be far less than the cumulative physician density of 0.08 per 1000 people [11], this may imply a higher work load for specialist physicians. Also, the absolute shortage of other public health personnel in Cameroon—as epitomized by a nurses’ density of 0.67 per 1000—is further complicated by the geographic distributional inequalities across the regions of the nation [12] and this can hinder optimal physician practice. Withal, the prevalence and correlates of burnout syndrome has not been assessed among Cameroonian doctors. This study will therefore inform public policy with respect to the training and distribution of physicians in the country, as well as shed light on the current state of physicians’ mental health in Cameroon.

## Main text

### General objective

To determine the prevalence and correlates of burnout syndrome amongst physicians in Cameroon.

### Specific objectives

1. To determine the prevalence of burnout syndrome amongst Cameroonian doctors.
2. To identify potential determinants of burnout among Cameroonian doctors.
3. To compare the prevalence and determinants of burnout among specialist physicians and general practitioners in Cameroon.

## Methods

### Population and design

#### Population

Physicians practicing in Cameroon (including general practitioners and specialist) will be included in the study. Doctors currently working in five out of Cameroon’s ten regions will be recruited by convenience sampling. The following regions will be included: Adamawa, Centre, Littoral, North West and South West regions. The selected regions were chosen due to easy accessibility and they cut across the major linguistic and religious divide of Cameroon. Amongst the five selected regions are three predominantly French-speaking regions and two English-speaking regions (of which one is a Muslim region and four are predominantly Christian). The choice of regions was mainly by convenience for investigators, and also to account for the diversity of Cameroon.

In the selected regions, rural/urban health institutions will be randomly selected and all consenting physicians will be recruited. Physicians working in the three different levels of health care delivery; the tertiary, the secondary, and the primary health services will be recruited as well as physicians in confessional and private hospitals.

#### Design

This will be a cross-sectional study that will run for a period of 2 months. Physicians working in the target regions will be visited in the hospital and informed consent sought. Once the physician signs the consent, a printed questionnaire will be handed to them, which they will be asked to fill in at their convenience and hand in. Doctors will receive either an English or a French copy of the questionnaire, depending on their preferred language of expression. All medical doctors will be recruited until the minimum sample size is attained.

#### Instrument

Data will be collected using a printed structured questionnaire, which comprises two sections. Section one will include: socio-demographic characteristics; work environment and leisure time activities. The following variables will be assessed: age; sex; marital status; presence of dependents; duration of medical practice; location of practice (rural vs urban); level of healthcare practice (primary vs secondary vs tertiary); number of patients seen; remuneration; number of hours of work; number of night calls; working relationship with colleagues; annual occupational leaves; average night’s sleep; practice of physical exercise; alcohol use; smoking; and history of any chronic disease.

Section two will assess burnout using the Maslach Burnout Inventory-Human Services Survey (MBI-HSS) [13], which is a reliable, widely used and a validated tool for assessment of burnout in African healthcare personnel [14]. The MBI-HSS is a self-administered questionnaire consisting of 22 statements on job-related feelings which the respondent answers according to their perceived frequency of occurrence, ranging from never to every day (on corresponding scale from 0 to 6). The three main subsets—emotional exhaustion; depersonalization and lack of personal achievement—are scored separately. Each of these tenets of burnout are further categorized according to severity (low, moderate or high level of burnout) [13].

#### Sampling

Physicians will be recruited by convenience sampling.

#### Sample size

Using the following formula [15]:

$$n = \frac{{Z^{2} P \left( {1 - P} \right)}}{{d^{2} }}$$

where n = sample size, Z = Z statistic for a level of confidence, p = expected prevalence or proportion (in proportion of one; 32%, p = 0.32) [16], and d = precision (in proportion of one; if 5%, d = 0.05). Z statistic (Z): for the level of confidence of 95%, which is conventional, Z value is 1.96. For a 95% confidence intervals (CI).

A minimum of *335 physicians* will be required for this study.

### Data management and statistical analysis

Data will be collected using a printed data collection form. Data entry will be done using EPI Info version 7 (CDC, Atlanta). To ensure accurate data entry, a random sample of 10% of the data will be crosschecked. Data back-up will be done daily and data analysis will be carried out using Epi info software.

Results will be presented as count (percentages), mean and standard deviation (SD) or median and 25th–75th percentiles as appropriate. The reliability and validity of the MBI-HSS will be determined by means of Cronbach alpha coefficients and factor analyses. Pearson correlation will be used to assess univariate associations between continuous variables. Multivariable logistic regressions will be used to identify determinants of burnout amongst doctors; odd ratio (OR) with 95% confidence intervals (CI) will be presented. A p value < 0.05 will be considered significant.

#### Objective one

*To determine the prevalence of burnout syndrome amongst Cameroonian doctors.* The three subscales of the MBI (emotional exhaustion; depersonalisation and personal accomplishment) will be used to define burnout. Accordingly, those who score high on the emotional exhaustion (score of 27 or higher) and/or depersonalization (score of 10 or higher) domains of burnout, will be considered to have manifestations of professional burnout [17].

#### Objective two

*To identify potential determinants of burnout among Cameroonian doctors.* Standard descriptive statistics and Chi square test or Mann–Whitney *U* test/two-sample Students *t* test procedures, as appropriate, will be used for univariate analyses to characterize and compare potential determinants such as: relationship status, sex, age, duration of practice, site of practice (rural vs urban), career level (specialist vs generalist).

#### Objective three

*To compare the prevalence and determinants of burnout among specialist physicians and general practitioners in Cameroon.*

Multivariate logistic regression analyses will be done for general practitioners and specialist physicians to identify socio-demographic and professional characteristics associated with burnout.

A p value < 0.05 will be considered statistically significant.

## Discussion

Mental health is largely a neglected field in Cameroon like in most developing countries. Similarly, data on the health providers’ health in Cameroon is sparse, although it is largely known that the quantity and quality of human resources for healthcare is a fair reflection of a country’s state of health.

Furthermore, studies have shown that public health workers expressed dissatisfaction with working and living conditions in Cameroon due to low remuneration, overwork burden, lack of professional autonomy, limited career advancement opportunities and lack of optimal equipment for smooth and effective practices in Cameroon [18]. All foregoing factors are likely at adversely affect physicians output and sense of wellbeing.

Generating the data on burnout syndrome amongst doctors in the various settings in Cameroon, will help mobilize researchers, practitioners and policy-makers on the potential burden of this rising health problem. This will ultimately lead to the consideration, development and implementation of policies to improve physicians’ health in Cameroon.

## Limitations

To the best of our knowledge, this is the first multicenter study to assess the prevalence and determinants of burnout in physicians in Cameroon. The limitations of this study will be inherent of its cross-sectional design; hence causality cannot be ascertained.

## Abbreviations

CI: confidence interval
ICD: International Classification of Diseases
MBI-HSS: Maslach Burnout Inventory-Human Services Survey
OR: odd ratio
SD: standard deviation
WHO: World Health Organization
